# Spontaneous Rupture of Pyometra

**DOI:** 10.1155/2013/298383

**Published:** 2013-08-19

**Authors:** Fatemeh Mallah, Tahere Eftekhar, Mohammad Naghavi-Behzad

**Affiliations:** ^1^Departments of Obstetrics and Gynecology, Valiasr Hospital, Tehran University of Medical Sciences, Tehran 1419733141, Iran; ^2^Medical Philosophy and History Research Center, Tabriz University of Medical Science, Tabriz 51368, Iran

## Abstract

Spontaneous perforation is a very rare complication of pyometra. The clinical findings of perforated pyometra are similar to perforation of the gastrointestinal tract and other causes of acute abdomen. In most cases, a correct and definite diagnosis can be made only by laparotomy. We report two cases of diffuse peritonitis caused by spontaneous perforated pyometra. The first case is a 78-year-old woman with abdominal pain for which laparotomy was performed because of suspected incarcerated hernia. The second case is a 61-year-old woman with abdominal pain for which laparotomy was performed because of symptoms of peritonitis. At laparotomy of both cases, 1 liter of pus with the source of uterine was found in the abdominal cavity. The ruptured uterine is also detected. More investigations revealed no malignancy as the reason of the pyometra.

## 1. Introduction

Pyometra is the collection of pus in the uterine cavity, and it is known as a rare condition [1]. The main cause of pyometra is occlusion of the cervical canal secondary to benign or malignant cervical or endometrial lesions and consequences of their treatments, cervicitis, after vaginal surgery, puerperal infection, and congenital cervical anomaly [2]. Spontaneous perforation of pyometra and subsequent diffuse peritonitis are also very rare with an incidence of about 0.01%–0.05% [1]. The correlation between pyometra and malignancies, risk of perforation, and high mortality rate make clinicians be aware of this disease especially in postmenopaused women presenting with acute abdomen [3, 4]. We report two women who were treated under a clinical diagnosis of diffuse peritonitis caused by spontaneously perforated pyometra without malignancy. 

## 2. Case Presentations

The first case is a 78-year-old multiparous woman who had Le Fort surgery five weeks ago because of procidentia and medical problems. She was admitted because of fever, vomiting, and diffused abdominal pain during the last 24 hours. She had epigastric hernia in her past medical history. The vital signs were stable. She had tender abdomen and normal pelvic examination. The laboratory tests included hemoglobin = 8.7 g/dL, BUN = 64 mg/dL, and creatinine = 1.1 mg/dL. Other tests were in normal ranges.

She was diagnosed with incarcerated hernia and then laparotomy was performed. At the laparotomy, 1 liter of pus arising from a perforated uterus was found in the abdominal cavity. The fundus of the uterus was found to have a perforation about 2 cm in diameter with purulent discharge. Abdominal hysterectomy and bilateral salpingo-oophorectomy was performed. Histopathological findings revealed severe inflammation and necrosis involving endometrium, myometrium, upper part of cervix, and right fallopian tube. She was discharged with good condition on the 20th postoperative day.

The second case is a 61-year-old multiparous menopaused woman who had history of treatment of pyelonephritis one week ago. The surgery which was performed was total abdominal hysterectomy and bilateral salpingo-oophorectomy. She was admitted because of fever, nausea, and diffused abdominal pain during the past 48 hours. Her gynecologic history was unremarkable. She had history of low abdominal pain for many days. She looked very ill with unstable vital signs (blood pressure = 90/50 mm Hg, body temperature = 38, and pulse rate = 120 beats/min) and also having no urine output. Her abdomen was distended with generalized tenderness and rebound tenderness. The laboratory tests were as follows: white blood cell count 21000 with 90% neutrophils, hemoglobin 10.2 g/dl, prothrombin time = 18 seconds, partial thromboplastin time = 40 seconds, INR = 2.1, C reactive protein = 102 mg/dL, BUN = 35 mg/dL, creatinine = 1.8 mg/dL, and platelet count = 137000.

She was diagnosed as having diffuse peritonitis due to rupture of ovarian abscess, and laparotomy was performed. At the laparotomy, 1 liter of pus arising from a perforated uterus was found in the abdominal cavity. The posterior wall of the uterus was found to have a perforation about 2 cm in the diameter with purulent discharge. Pathological investigation of the surgical specimen revealed endometritis and myometritis of the uterus and bacterial aggregation and fat necrosis, but there was no evidence of malignancy. She was discharged on the 30th postoperative day after treatments.

## 3. Discussion

Accumulation of purulent material in the uterine is termed as pyometra. It is an uncommon condition occurring mainly in elderly postmenopausal females and due to impaired drainage of uterine cavity. Impaired drainage conditions include cervical canal stenosis, benign or malignant cervical lesions, and surgical complications [5]. Although atrophic endometrium is a common cause of pyometra, perforation is usually seen in the presence of serious causes such as cervical or endometrial carcinoma or a forgotten IUCD. Malignant disease is present in 35% of cases [6]. The classic triad of pyometra is lower abdominal pain, purulent vaginal discharge, and postmenopausal bleeding. However, more than 50% of all cases are asymptomatic [7].

Spontaneous rupture of pyometra is extremely rare and the incidence is 0.01–0.05% with only 28 cases reported and indexed in the English literature [6, 8–10]. All patients were postmenopausal women like our cases except for 34- and 41-year-old women. The most common symptom was abdominal pain, and some of them had fever, vomiting, and nausea at the time of presentation. The common preoperative diagnosis was generalized peritonitis (50%), perforation of gastrointestinal tract (40%), and pneumoperitoneum (30%). The treatment of pyometra rupture is immediate laparotomy, peritoneal lavage and drainage, and total hysterectomy. In our first case, a 78-year-old woman with fever, vomiting, and abdominal pain had primarily been diagnosed as incarcerated hernia. Second one was 61-year-old woman with fever, nausea, and diffuse abdominal pain that had been diagnosed as peritonitis. Laparotomy was performed, and perforated uterine was detected. In addition, 1 liter of pus was collected from the abdominal cavity in each case. Histopathological studies after hysterectomy revealed inflammation of endometrium and myometrium. There was no evidence of malignancy in both cases. Therefore, the most probable cause of pyometra was postmenopausal changes and stenosis of cervix.

From a report of Saha et al., 73% of the nonmalignancy cases had favorable prognoses for survival, whereas only 33% of malignancy cases did so [9]. Considering nonmalignant reason of the pyometra, our both cases had proper recovery periods and were discharged with good condition.

## 4. Conclusion

Although spontaneous rupture of pyometra is rare, it should be kept in mind as a differential diagnosis in postmenopausal women presenting with acute abdomen, because correct diagnosis, early intervention, intensive pre- and postoperative care, and proper treatment can reduce morbidity and mortality.
